# Cost-Effectiveness of Short Course of Ceftazidime/Avibactam for *K. pneumoniae*-KPC Bloodstream Infections in Italy

**DOI:** 10.3390/microorganisms11051102

**Published:** 2023-04-23

**Authors:** Ilaria De Benedetto, Nour Shbaklo, Costanza Vicentini, Carla Maria Zotti, Francesco Giuseppe De Rosa, Silvia Corcione

**Affiliations:** 1 Department of Medical Sciences, Infectious Diseases, University of Turin, 10126 Turin, Italy; 2 Department of Public Health and Paediatrics, University of Turin, 10126 Turin, Italy; 3 Infectious Diseases Unit, Cardinal Massaia Hospital, 14100 Asti, Italy; 4 School of Medicine, Tufts University, Boston, MA 02111, USA

**Keywords:** *Klebsiella pneumoniae*, blood-stream infection, ceftazidime-avibactam

## Abstract

Background: Evidence has shown that short courses of antibiotic therapy are at least as effective as long courses with better clinical outcomes. CAZ/AVI has demonstrated its clinical efficacy in treating *K. pneumoniae*-KPC infections. Methods: We conducted an analysis based on the real-life data of our ten years retrospective cohort to assess the cost-effectiveness and cost-utility of a short course of CAZ/AVI plus source control compared to a long course plus source control. A Markov model was structured. Patient transition between health states was modeled, each transition has a probability, and each state has a cost and a utility. Incremental cost-effectiveness ratios (ICERs) were obtained by dividing the difference in costs by the difference in utilities between the two courses. Input parameter uncertainty was investigated through sensitivity analysis. We launched 1000 Monte Carlo simulations by iteratively perturbing variables within estimated variation ranges, obtaining an ICER result for each simulation. Results: In the first model (old appropriate treatment), a short course of treatment was associated with reduced costs per patient per year of €4818.60 and reduced effects (0.10 QALYs), compared to a long course. In the CAZ/AVI model, the short course was associated with increased costs of €1297.9 and with increased effects (0.04 QALYs), resulting in an ICER of €32,317.82 per QALY gained, below the WTP threshold of €40,000. Conclusions: Our findings highlight additional evidence regarding the cost-effectiveness of CAZ/AVI for policy-makers. We outline that CAZ/AVI could be cost-effective compared to old appropriate antibiotic therapies for KPC-Kp BSI.

## 1. Introduction

Several studies have compared the impact of a short versus long course of antimicrobial therapy aiming to limit adverse effects, promote early treatment discontinuation and discharge, and avert the ecological pressure leading to the emergence of antimicrobial resistance [1,2,3,4,5,6,7,8]. Evidence has shown that short courses of antibiotic therapy are at least as effective as long courses with better clinical outcomes, fewer adverse events in several settings—also in pneumonia—and may reduce the emergence of resistant organisms compared to a prolonged course. In a nationwide cohort of patients with sepsis [3], short-course administration contributed to a decrease in medical costs and 28-day mortality. Moreover, in a recent meta-analysis including 1186 patients with bloodstream infections sustained by Gram-negative bacteria, no differences in mortality and clinical outcomes were demonstrated between a 7- and 14-day course of antibiotic treatment [7]. Nonetheless, the proportion of included multidrug-resistant pathogens, which are notoriously considered difficult to treat because of the limited treatment options, ranges from 8 to 18%.

Ceftazidime/avibactam (CAZ/AVI) is a relatively new third-generation cephalosporin combined with a non-β-lactam-β-lactamase inhibitor which inhibits class A enzymes, including extended-spectrum beta-lactamase (ESBLs), *Klebsiella pneumoniae* carbapenemase (KPC), class C and some OXA β-lactamases, but it has no activity against metallo-β-lactamases (MBLs). It was approved by the Food and Drug Administration (FDA) and the European Medicines Agency (EMA) for treating complicated urinary tract infections (cUTIs), complicated intraabdominal infections (cIAIs), hospital-acquired pneumonia (HAP), ventilator-acquired pneumonia (VAP), and other infections due to Gram-negative bacteria with limited treatment options [9,10]. In a pivotal trial including cUTIs caused by ESBLs, CAZ/AVI and doripenem showed similar clinical cure rates [11]. Despite limited data on the management of carbapenem-resistant *Enterobacterales* infections, several observational studies after its commercialization have shown an association with CAZ/AVI therapy and lower mortality rates compared to previously used drug regimens including meropenem, colistin, tigecycline, or their combinations [12,13,14,15,16,17].

In previous work by our group [17], source control and appropriate empirical therapy with CAZ/AVI emerged as protective factors for mortality in a large cohort of *K. pneumoniae*-KPC bloodstream infections. Since CAZ/AVI has demonstrated its clinical efficacy and its use is now consolidated for the treatment of *K. pneumoniae*-KPC infections, the economic benefits of this drug have been assessed through cost-effectiveness and cost-utility analyses comparing CAZ/AVI to the previously available treatments. Tichy et al. [18] have demonstrated based on the REPROVE trial data on HAP/VAP [19] that CAZ/AVI followed by colistin + high-dose meropenem versus meropenem followed by colistin + high-dose meropenem provided a better clinical cure rate and gains in the number of life-years (+0.195) and QALYs (+0.350) per patient with an estimated net incremental total cost of €1254 ($1401) per patient. The estimated incremental cost-effectiveness ratio was €3581 ($4000) per QALY gained, below the willingness-to-pay threshold of €30,000 ($33,507) per QALY in Italy. Similarly, Kongnakorn et al. [20] have demonstrated based on the RECLAIM [21] trial data on cIAI that CAZ/AVI plus metronidazole followed by a colistin + tigecycline + high-dose meropenem combination compared to ceftolozane/tazobactam plus metronidazole followed by colistin + tigecycline + high-dose meropenem and meropenem followed by colistin + tigecycline + high-dose meropenem had better clinical outcomes with higher cure rates and higher QALYs gained per patient (4.021 vs. 3.982; 4.019 vs. 3.960, respectively). The incremental cost-effectiveness ratio of CAZ/AVI was €4099 and €15,574 per QALY gained versus each comparator, respectively, below the willingness-to-pay threshold.

We conducted an analysis based on the real-life data of our ten years retrospective cohort to assess the cost-effectiveness and cost-utility of a short course (<7 days) of CAZ/AVI plus source control compared to a long course (≥7 days) of CAZ/AVI plus source control. Then we further compared the CAZ/AVI therapy versus a short (<7 days) or long course (≥7 days) of old appropriate treatment in bloodstream infections sustained by *K. pneumoniae*-KPC.

## 2. Methods

The aim of our analysis was to investigate whether the results of the recent meta-analysis by Turjeman et al. about the duration of antibiotic treatment for Gram-negative bacteremia [7] would translate into benefits from a cost-utility perspective in a highly endemic setting for *K. pneumoniae*-KPC such as Italy. We built a model incorporating data from the literature and retrospectively collected data from our tertiary care center, to determine the difference in costs of short (<7 days) vs. long-course (≥7 days) (standard of care) of targeted antibiotic therapy and the difference in effects in terms of quality-adjusted life years (QALYs). Two models were built, comparing (1) short vs. long course of old appropriate treatment and (2) short vs. long course of CAZ/AVI either with source control for the treatment of bloodstream infections sustained by *K. pneumoniae*-KPC (KPC-Kp BSI).

### 2.1. Model Design

**Figure 1:**
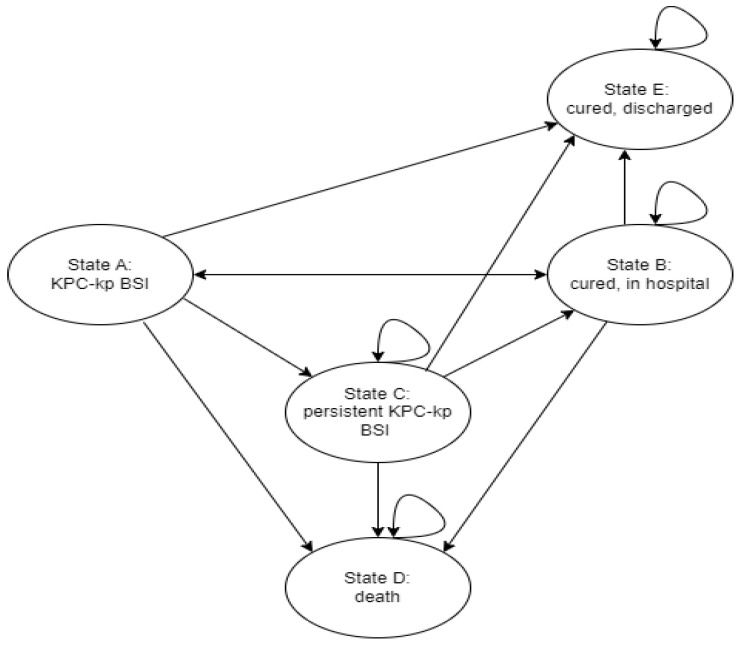
Markov model structure.

A Markov model structure was chosen as it allows us to consider recurrent events. Figure 1 summarizes the model structure and considered health states. Patients transition between health states, each transition has a probability, and each state has a cost and a utility. Transitions occur over a cycle and in our model cycle length was set to 30 days. Two tunnel states—states C and B—were added to consider the time spent in health state A, as we assumed length of infection—and consequently, the need to prolong hospitalization—would affect the probability of dying, and the different cost and utilities associated with states B and E. Models were run for 12 cycles (1 year).

Patients begin each cycle in state A: KPC-Kp BSI. Patients then transition to four other states: state B (cured, in hospital), state E (cured, discharged), state C (persistent KPC-Kp BSI, defined as microbiological failure), and state D (death). Patients in state C can remain in state C, or transition towards states D, B, or E. Patients in state B can remain in state B, return to state A (relapse), or transition to states D and E. States D and E are absorbing states as mortality and recurrent infections after discharge were not incorporated in the model.

### 2.2. Input Parameters

*Probabilities.* Point estimates were obtained from the studies included in the meta-analysis by Turjeman et al. [7] and from data collected in our center (full details on demographic and clinical characteristics of included patients are available as Supplementary Table S1). Mortality estimates were extracted from our cohort, as we considered they would be more representative of patients with KPC-Kp BSI. For modelling reasons, even though the attributable death rate of KPC BSI was unknown, we assumed deaths caused by BSI were all deaths occurring with ongoing antibiotic therapy, due to the severe nature of KPC-Kp BSI. Based on the variance between estimates, standard deviation estimates were chosen. Probabilities B to E were estimated as the inverse of the median length of stay in months following cure and interruption of antibiotic treatment. Probabilities A to B, B to B, and C to B were obtained through mathematical extrapolation. Table 1 reports probability estimates, variance ranges, and references for data sources.

*Costs.* Costs were evaluated from the hospital perspective and were obtained from our setting. We did not apply discounting as models were run for 12 months. We evaluated the following costs: costs associated with a hospital stay, distinguishing admission to intensive care units (ICUs) and regular wards, and drug costs. The latter were considered only in the CAZ/AVI model, as drug costs for older antibiotics are negligible compared to costs associated with a hospital stay. Costs were assigned to states A, B, and C, whereas we did not consider any costs for states D and E (i.e., we did not consider costs associated with treatments post-discharge).

To evaluate costs associated with a hospital stay, we evaluated the proportion of patients in our cohort in states A, B, and C who were first diagnosed in ICUs vs. regular wards (Supplementary Table S1). The cost of a month of stay was obtained from hospital management: €52,200 in ICU (€1740 per day) and €14,280 in regular wards (€476 per day).

The cost of a daily dose of ceftazidime-avibactam was obtained from our hospital pharmacy and resulted in €173.16. We assumed a short course of treatment to be 7 days, whereas we considered the actual length of treatment of patients in our cohort receiving CAZ/AVI for a long course: median of 11 days (interquartile range, IQR 10–14). Table 2 reports detailed costs per health states A, B, and C.

*Utilities*. Utilities were obtained from the study by Koukoubani et al. [22], which evaluated QALYs at six months and one year among patients with healthcare-associated infections due to antimicrobial-resistant pathogens (98% of which were BSIs and 56% due to kp) in a Greek population. We assigned a utility value of 0.070 ± 0.102 for states A and C (acute ongoing infection), and of 0.179 ± 0.263 for states B and E (recovery from infection). A value of 0 was assigned to state D.

### 2.3. Cost-Utility Analysis

*Base-case.* Base-case results were obtained by inputting point estimates for each parameter (transition probabilities, costs, and utilities). Incremental cost-effectiveness ratios (ICERs) were obtained by dividing the difference in costs by the difference in utilities between short vs. long course treatment obtained in both models. We set willingness to pay (WTP) thresholds at €30,000 and €40,000 per QALY [23].

*Deterministic one-way sensitivity analysis.* Input parameter uncertainty was investigated through sensitivity analysis. We varied the following transition probabilities according to ranges reported in Table 1: the probability of persistent BSI (microbiological failure, transition A to C) and the probability of relapse after microbiological cure (transition B to A). Costs were varied across ranges reported in Table 2: we assumed costs of hospital stay and drug costs would not vary, but assigned a standard deviation of 0.05 to the proportion of patients diagnosed in ICUs and considered the interquartile range of CAZ/AVI length of treatment for long course. For QALYs, we applied the ranges estimated by Koukoubani et al. [22].

*Probabilistic sensitivity analysis.* We launched 1000 Monte Carlo simulations by iteratively perturbing variables within estimated variation ranges, obtaining an ICER result for each simulation. Each ICER was calculated by randomly selecting values within variable ranges simultaneously. We assumed a beta distribution for transition probabilities and QALYs and a gamma distribution for costs. We then calculated the proportion of ICERs under both WTP thresholds and calculated 95% confidence intervals (CIs) for both proportions through bootstrap resampling (1000 iterations). We used Microsoft Excel 2016 to build models and performed statistical analyses using IBM SPSS version 28.0.

## 3. Results

*Base-case.* Table 3 shows the reported base-case results. In the first model (old appropriate treatment), a short course of treatment (<7 days) was associated with reduced costs per patient per year of €4818.60 and reduced effects (0.10 QALYs), compared to a long course of treatment (≥7 days). In the CAZ/AVI model, the short course was associated with increased costs of €1297.9 and with increased effects (0.04 QALYs), resulting in an ICER of €32,317.82 per QALY gained, below the WTP threshold of €40,000.

**Figure 2:**
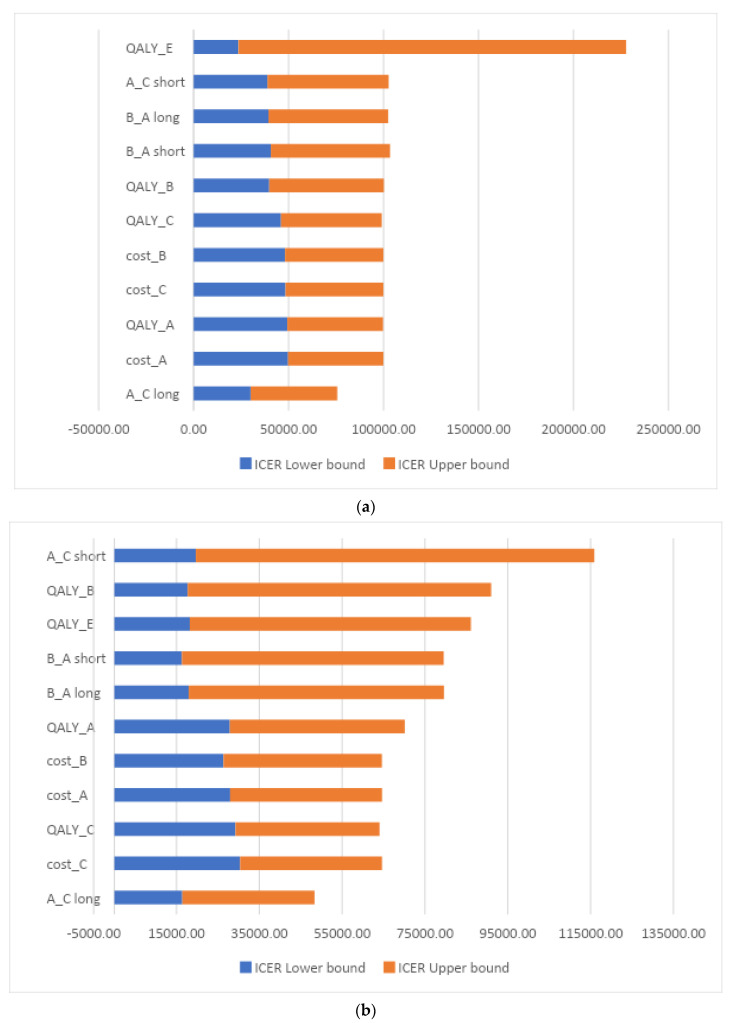
Tornado diagram—Incremental cost-effectiveness ratios (ICERs) resulting from one-way deterministic sensitivity analysis comparing short vs. long course antibiotic treatment. (**a**) Old appropriate treatment model. (**b**) Ceftazidime/avibactam model.

*Deterministic sensitivity analysis.* Figure 2 depicts result ranges for ICERs obtained at one-way deterministic sensitivity analyses comparing short vs. long course antibiotic treatment. In the first model (Figure 2a), ICERs ranged from €23,611.49 to €204,124.48. The greatest variation was found by varying QALY estimates for state E. As shown in Figure 2b, the second model was more robust to input parameter uncertainty, with ICERs ranging from €16,297.79 to €96,243.17.

**Figure 3:**
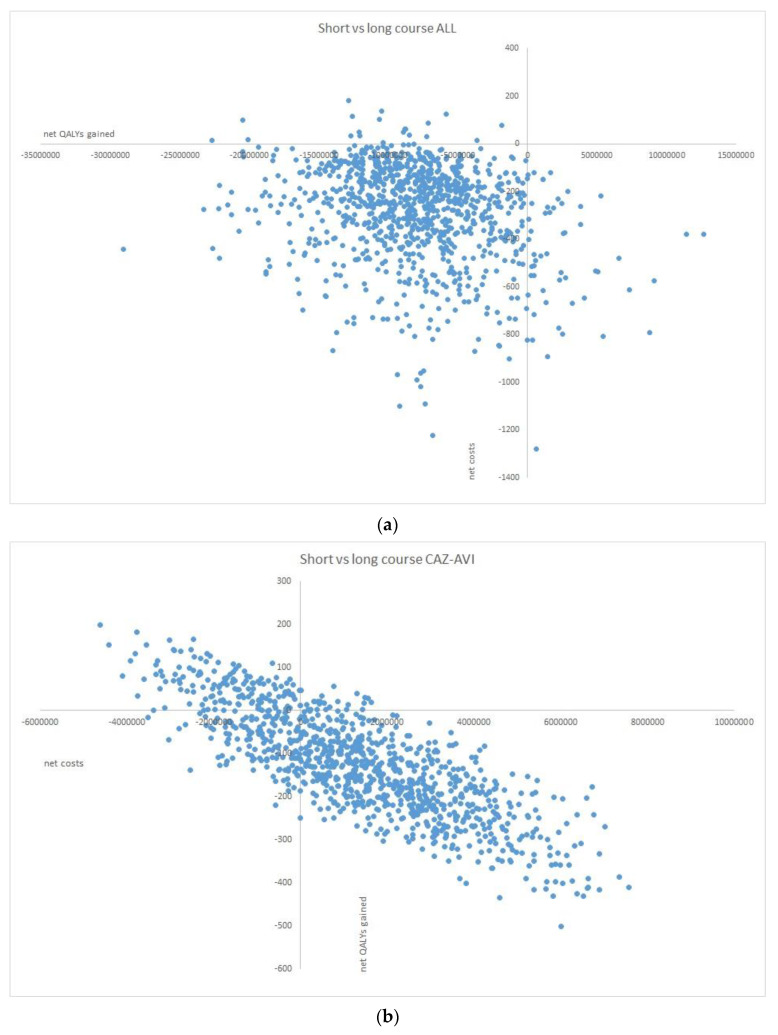
Net costs and net quality-adjusted life years (QALYs) of comparing short vs. long course antibiotic treatment, resulting in incremental cost-effectiveness ratios (ICERs), at 1000 Monte Carlo simulations. (**a**) Old appropriate treatment model. (**b**) Ceftazidime/avibactam model.

*Probabilistic sensitivity analysis*. ICER results for 1000 Monte Carlo simulations are shown in Figure 3. In the first model, short course vs. long course treatment was associated with a proportion of ICERs below the WTP threshold of €30.000 of 51% (95% CI 48–54), whereas the proportion of ICERs below the WTP threshold of €40.000 was 62.5% (95% CI 59.4–65.52). In the second model, the proportion of ICERs below the WTP threshold of €30.000 was 94.4% (95% CI 92.8–95.6), whereas the proportion of ICERs below the WTP threshold of €40.000 was 96% (95%CI 94.2–96.7).

## 4. Discussion

Several studies supported the use of short antibiotic therapy in different clinical conditions, showing no differences in mortality and improving adverse effects, and promoting early discharge. Our analysis aimed to investigate whether these results emerging from growing literature would translate into benefits from a cost-utility perspective in a highly endemic setting for *K. pneumoniae*-KPC such as Italy. Moreover, potential obstacles in the use of newer antibiotics such as CAZ/AVI might be represented by costs despite the demonstrated efficacy in RCTs and retrospective studies compared to old appropriate treatments with an approximate value of $7500–$15,000 for a 7–14-day therapy [13,16,23].

For this reason, we conducted a cost-utility analysis comparing short vs. long courses of CAZ/AVI or old appropriate antibiotic regimens in our real-life cohort of patients with KPC-Kp BSI in a tertiary hospital in Northern Italy. The results of our analysis show that a short course of old antibiotic treatment is not cost-effective compared to long course treatment, but CAZ/AVI short course treatment could be cost-effective compared to a long course treatment. This finding might support the consideration of a short course of CAZ/AVI as a first-line option in KPC-Kp bloodstream infections, discouraging the use of combinations of old drugs both for the well-known clinical inferiority, for carbapenem-sparing and for cost-saving in the setting of stewardship interventions and optimization of resources [9,10,11,12,13,14,15,16,17,18].

In a similar base case analysis published by Simon et al. [24], CAZ/AVI was also cost-effective as a treatment for CRE bacteremia. It was suggested that it increased both costs and QALYs but accounted for an acceptable cost-effectiveness ratio ($95,000/QALY) upon U.S. health willingness-to-pay standards. The study showed that using CAZ/AVI as first-line treatment for CRE pneumonia and bacteremia, while sparring colistin, could result in gains of 2939 life-years and 2070 QALYs. Simon et al. [24], demonstrated that the ICER increased to $100,000/QALY only and $150,000/QALY when costs exceeded $35,600 and $144,000, respectively. Moreover, health-care associated costs and quality of life after surviving a CRE infection are highly important variables in cost-effectiveness models [25]. Limited data are available on the long-run health and economic concerns of CRE infections, but around 50% of patients with CRE infection or colonization are transferred to long-term care facilities [26]. In addition, readmission increases costs and adverse events after CRE infections, with a rate of up to 20% in patients with CRE infections [27]. According to mortality rates in patients treated with colistin published by Shields et al. [13] (30%) and van Duin et al. [17] (32%), the ICER did not exceed $100,000/QALY. Since van Duin et al. [16] reported a higher probability of home discharge for patients treated with CAZ/AVI than colistin [16], CAZ/AVI cost-effectiveness could be even more likely.

Our analysis has several important limitations that should be considered when interpreting results. Due to the retrospective nature of the analyzed cohort, potential biases could be possible such as recall bias and missing data, the lack of a severity score such as SOFA, and the possible change of treatments and approaches across 10 years of observation. As with any model, our analysis was limited by the validity of our assumptions and the accuracy of our estimates. Regarding assumptions underlying our model, as stated in the Methods section two tunnel states were added to consider the duration of infection, as we assumed this would affect the length of hospital stay and the probability of dying. We incorporated in our model what we considered were the most relevant elements of clinical pathways and aimed to avoid unnecessary complexity, and therefore we did not incorporate the full spectrum of possible health states and outcomes for patients with KPC-Kp BSI, in particular, post-discharge events were not considered. Concerning input parameters, an important limitation of this study is the limited availability of transition probability estimates, in particular for mortality (as stated in the methods section). However, we extracted data from studies included in a recent meta-analysis and performed sensitivity analyses over broad ranges. Further, we applied mortality estimates from what we expected would be the worst-case scenario in terms of patient case-mix (long course treatment, old appropriate treatment), therefore we can suppose results for short course treatment are conservative estimates, in particular for the CAZ/AVI model. Costs estimates are limited by having been obtained by a single center, and could have limited representativeness in countries without single-payer health systems. QALY estimates were obtained from a single Greek study [28], however, the clinical characteristics of included patients, as well as the broader epidemiological context [27], can be considered comparable to our setting.

To conclude, our aim was to provide a preliminary assessment of short course treatment for patients with KPC-Kp BSI, which are difficult to include in randomized clinical trials, in order to help guide future clinical investigations. Our findings highlight additional evidence regarding the cost-effectiveness of CAZ/AVI for policy-makers. We outline that CAZ/AVI could be cost-effective compared to old appropriate antibiotic therapies for KPC-Kp BSI. Our model with input parameters from a real-life cohort of KPC-Kp BSI patients and published data could be transferred to other similar settings with different willingness-to-pay thresholds to assess the cost-effectiveness of using up-to-date antibiotic regimens, such as CAZ/AVI. Future research on CAZ/AVI use in practice for CRE infections would aid in refining conclusions about the economic impact of CAZ/AVI.

## Figures and Tables

**Table 1:** Input parameters: transition probabilities.

		Short Course (<7 Days)		Long Course (≥7 Days) Old Appropriate Treatment		Long Course (≥7 Days) CAZ/AVI	
Transition	Probability description	Literature estimates	Point estimate of probability (estimated SD)	Literature estimates	Point estimate of probability (estimated SD)	Literature estimates	Point estimate of probability (estimated SD)
P(A to C)	Probability of persistent KPC-Kp BSI (microbiological failure)	8/110 [4] 3/169 [6]	0.039 (0.05)	12/122 [4] 2/165 [6] 1/31 [19]	0.047 (0.05)	0/14 [19]	0 (0.05)
P(A to D)	Probability of death due to primary KPC-Kp BSI		*	4/31 [19]	0.129 (0.05)	2/14 [19]	0.143 (0.05)
P(A to E)	Probability of cure and discharge within one month after primary KPC-Kp BSI		*	2/31 [19]	0.065 (0.05)	1/14 [19]	0.071 (0.05)
P(A to B)	Probability of cure without discharge within one month after primary KPC-Kp BSI	Mathematical extrapolation: 1–P(A to C)–P(A to D)–P(A to E)					
P(B to A)	Probability of relapse after microbiological cure	7/108 [4] 1/169 [6] 8/306 [5]	0.027 (0.02)	6/121 [4] 2/165 [6] 8/298 [5] 3/31 [19]	0.031 (0.02)	1/14 [19]	0.071 (0.02)
P(B to D)	Probability of death (in hospital) after cure		*	0/31 [19]	0 (0.05)	0/14 [19]	0 (0.05)
P(B to E)	Probability of being discharged after 1 month following microbiological cure		*	1/Median LOS in months: 3.0 (IQR 2.54–4.49) [19]	0.333 (0.223–0.393)	1/Median LOS in months: 2.93 (IQR 2.37–3.07) [19]	0.341 (0.326–0.423)
P(B to B)	Probability of remaining in hospital after cure	Mathematical extrapolation: 1–P(B to A)–P(B to D)–P(B to E)					
P(C to C)	Probability of persistent KPC-Kp BSI (microbiological failure) over 1 month		*	0/5 [19]	0 (0.05)		*
P(C to D)	Probability of death due to persistent KPC-Kp BSI over 1 month		*	1/5 [19]	0.2 (0.05)		*
P(C to E)	Probability of cure and discharge within one month after persistent KPC-Kp BSI		*	1/5 [6]	0.2 (0.05)		*
P(C to B)	Probability of cure without discharge within one month after persistent KPC-Kp BSI	Mathematical extrapolation: 1–P(C to C)–P(C to D)–P(C to E)					

* Assumed equivalent to long course—old appropriate treatment. IQR: interquartile range; KPC-Kp BSI: bloodstream infections sustained by *K. pneumoniae*-KPC; LOS: length of stay; SD: standard deviation.

**Table 2:** Input parameters: costs estimates and ranges in € per health states A, B, and C.

Health State	Old Appropriate Treatment Model *	CAZ/AVI Model	
		Short Course *	Long Course **
A	21,619.36 (19,721.52–23,513.52)	22,831.48 (20,933.64–24,725.64)	23,524.12 (21,626.28–25,418.28)
B	23,308.57 (21,408.96–25,200.96)	23,308.57 (21,408.96–25,200.96)	23,308.57 (21,408.96–25,200.96)
C	29,448 (27,552–31,344)	30,660.12 (28,764.12–32,556.12)	31,352.76 (29,456.76–33,248.76)

* range based on an assumed estimated standard deviation of the proportion of patients diagnosed in intensive care units (ICUs) of 0.05; ** range based on an assumed estimated standard deviation of the proportion of patients diagnosed in ICUs of 0.05 and over length of therapy interquartile range.

**Table 3:** Base case cost-utility analysis results: total costs, total quality-adjusted life years (QALYs), differences in costs and utilities, and incremental cost-effectiveness ratios (ICERs).

	Old Appropriate Treatment Model			CAZ/AVI Model		
	Short Course (<7 Days)	Long Course (≥7 Days)	Delta	Short Course (<7 Days)	Long Course (≥7 Days)	Delta
Costs per patient, €	78,121.88	82,940.49	−4818.60	79,459.61	78,161.71	1297.9
QALYs per patient	1.9	2	−0.10	1.9	1.86	0.04
ICER			49,959.59			32,317.82

## Data Availability

Data will be available upon justified request.

## Supplementary Materials

The following are available online at https://www.mdpi.com/article/10.3390/microorganisms11051102/s1, Table S1: Patient characteristics on 30-day mortality; Table S2: Patient characteristics based on ceftazidime-avibactam therapy vs. standard of care (SOC).
