# Supplementary material for: Cost-Effectiveness of Short Course of Ceftazidime/Avibactam for K. pneumoniae-KPC Bloodstream Infections in Italy
Source: Microorganisms. 2023 Apr 23;11(5):1102. doi: 10.3390/microorganisms11051102 (PMC10224085; doi:10.3390/microorganisms11051102)
Supplement: Supplementary file 1 [file microorganisms-11-01102-s001.zip › microorganisms-2326211-supplementary.pdf]

Table S1: Patient characteristics on 30-day mortality

Variable	Alive on day 30 N= 329 (62.7)	Dead on day 30 N=195 (37.2)	Total N=524 (100)	p-value
I. Demographics				
**Gender:**				
Male	209 (63.9)	118 (60.5)	327 (62.6)	
Female	118 (36.1)	77 (39.5)	195 (37.4)	0.437
Age	61.4 ± 15.2	64.6 ± 14	62 ± 14	**0.010**
**Ward:**				0.350
• **Medical ward**	119 (36.4)	63 (32.3)	182 (34.9)	
• **Surgical ward**	115 (35.2)	65 (33.3)	180 (34.5)	
• **Intensive care unit**	93 (28.4)	67 (34.4)	160 (30.7)	
ICU vs. other wards	94 (28.7)	67 (34.4)	161 (30.8)	0.179
Length of stay	53 ± 31	30 ± 20	41 ± 25	**0.005**
II. Clinical course				
**Comorbidities:**				
• Congestive heart failure	70 (21.5)	60 (30.8)	130 (25)	**0.018**
• Chronic lung disease	63 (19.3)	51 (26.2)	114 (21.9)	0.068
• Liver disease	26 (8.0)	23 (11.8)	49 (9.4)	0.148
• Chronic Kidney Disease	72 (22.0)	54 (27.7)	126 (24.1)	0.143
• Metastatic malignancy	19 (5.8)	14 (7.2)	33 (6.3)	0.534
• Hematologic malignancy	29 (8.9)	23 (11.8)	52 (10.0)	0.280
• Diabetes mellitus	64 (20.4)	44 (23.3)	108 (21.5)	0.443
• Neutropenia	15 (4.8)	8 (4.2)	23 (4.6)	0.777
• SOT	42 (12.8)	22 (11.3)	64 (12.3)	0.588
• HIV	0 (0.0)	2 (1.0)	2 (0.4)	0.139
**Surgery during stay:**				
• Surgery other than abdominal	96 (30.9)	65 (34.4)	161 (32.2)	0.385
• Abdominal surgery	77 (24.8)	37 (19.6)	114 (22.8)	
Charlson score	3.3 ± 2,1	3.8 ± 2.3	4 ± 2	**0.028**
**Invasive Procedures:**				
• CVC	272 (83.2)	178 (91.3)	450 (86.2)	**0.009**
• Mechanical Ventilation	143 (43.7)	126 (64.6)	269 (51.5)	**<0.001**
• Urinary catheter	244 (46.5)	164 (31.2)	408 (77.8)	**0.013**

• ECMO	16 (4.9)	26 (13.3)	42 (8.1)	**<0.001**
• Hemodialysis	21 (6.4)	28 (14.4)	49 (9.4)	**0.006**
• CVVH	41 (12.6)	54 (27.7)	95 (18.2)	**<0.001**
**Possible source:**				**<0.001**
• Respiratory	52 (16.4)	48 (26.1)	100 (20)	
• CVC	82 (25.9)	36 (19.6)	118 (23.6)	
• Urinary catheter	35 (11)	5 (2.7)	40 (8)	
• Intraabdominal	23 (7.3)	13 (7.1)	36 (7.2)	
• Multiple sites	23 (7.3)	13 (7.1)	36 (7.2)	
• Unidentified	85 (26.8)	67 (36.4)	152 (30.2)	
Source control	114 (36)	35 (18.9)	149 (29.7)	**<0.001**
III. Microbiological characteristics:				
Time to KPC BSI from admission	29.8 ± 33	26.4 ± 24	28 ± 30	0.825
KPC colonization	179 (57)	97 (51.3)	276 (54.9)	0.215
**KPC in samples:**				
• Respiratory	114 (34.9)	76 (39.0)	190 (36.4)	0.345
• Urinary	90 (27.4)	35 (17.9)	125 (23.9)	**0.014**
• Drainage	44 (13.5)	23 (11.8)	67 (12.8)	0.583
**MDR Coloniztion:**	21 (6.4)	12 (6.2)	33 (6.3)	0.903
• A-baumannii	11 (3.4)	9 (4.6)	20 (3.8)	0.471
• VRE	7 (2.1)	2 (1.0)	9 (1.7)	0.495
• ESBL	3 (0.9)	2 (1.0)	5 (1.0)	>0.99
Clostridium difficille	9 (2.8)	4 (2.1)	13 (2.5)	0.775
**Polymicrobial BSI:**	29 (8.9)	18 (9.2)	47 (9)	0.889
• P.aeruginosa	1 (0.3)	5 (2.6)	6 (1.1)	0.029
• VRE	5 (1.5)	4 (2.1)	9 (1.7)	0.733
• ESBL	9 (2.8)	3 (1.5)	12 (2.3)	0.549
• MRSA	5 (1.5)	0	5 (1.0)	0.163
• Candida	2 (0.6)	5 (2.6)	7 (1.3)	0.108
**Post BSI:**				
• MRSA	1(0.3)	0(0.0)	1(0.2)	>0.99
• Candidemia	4(1.2)	3(1.5)	7(1.3)	0.715
• CDI	5(1.5)	2(1.0)	7(1.3)	>0.99
General Combination	2 ± 1	2 ± 1	2 ± 1	0.255
**Empiric therapy:**	217 (66.6)	151 (77.8)	368 (70.8)	**0.006**
• Empiric therapy appropriate	110 (48.7)	72 (46.5)	182 (47.8)	0.670
• Days Empiric therapy	10 ± 9	10 ± 8	11 ± 10	0.438
• Combination empiric	1 ± 1	2 ± 1	2 ± 1	**0.003**
• Meropenem	106 (50.7)	77 (52.4)	183 (51.4)	0.757

• Amikacin	21 (10)	9 (6.2)	30 (8.4)	0.200
• Tigecycline	61 (29.2)	56 (38.4)	117 (33)	0.071
• Colistin	40 (19.2)	31 (21.1)	71 (20)	0.666
• Piperacillin tazobactam	66 (31.6)	69 (46)	135 (37.6)	**0.005**
• Bactrim	11 (5.3)	8 (5.5)	19 (5.4)	0.937
• Fosfomycin	12 (5.7)	9 (6)	21 (5.8)	0.900
• Ceftazidime-avibactam	14 (6.5)	6 (4.1)	20 (5.5)	0.320
**Targeted therapy:**	236 (72.8)	111 (57.8)	347 (67.2)	**<0.001**
• Days Targeted Therapy	17 ± 14	9 ± 7	15 ± 13	**<0.001**
• Combination targeted	2 ± 1	2 ± 1	2 ± 1	0.32
• Meropenem	103 (46.2)	54 (50.9)	157 (47.7)	0.420
• Amikacin	23 (10.5)	8 (7.5)	31 (9.5)	0.402
• Tigecycline	119 (54.1)	74 (69.8)	193 (59.2)	**0.007**
• Colistin	65 (29.1)	47 (43.9)	112 (33.9)	**0.008**
• Gentamycin	103 (45.8)	47 (44.3)	150 (45.3)	0.806
• Ertapenem	29 (13.1)	7 (6.7)	36 (11)	0.082
• Bactrim	26 (11.8)	7 (6.7)	33 (10.1)	0.154
• Fosfomycin	33 (14.6)	14 (13.1)	47 (14.1)	0.710
• Ceftazidime-avibactam	59 (25)	22 (19.3)	81 (23.1)	0.236
Monotherapy	99 (30.7)	51 (27.1)	150 (29.4)	0.387
On-time therapy	197 (61.2)	117 (61.3)	314 (61.2)	0.986
KPC reoccurence	27 (8.2)	2 (1)	29 (5.5)	**<0.001**
**7-day nephrotoxicity:**				**<0.001**
• Nephrotoxicity	33 (17.7)	49 (39.8)	82 (26.5)	
• Acute renal failure at baseline or death before 7 days	42 (22.6)	36 (29.3)	78 (25.2)	
Time to death	57.7 ± 33	10 ± 8.2	21 ± 26	**<0.001**
In-hospital mortality	50 (15.2)	175 (89.7)	225 (43)	**<0.001**

ICU: intensive care unit, SOT: solid organ transplant, HIV: Human immunodeficiency virus, CVC: central venous catheter, ECMO: extracorporeal membrane oxygenation, CVVH: continuous veno-venous hemofiltration , KPC: Klebsiella pneumoniae carbapenemase, VRE: vancomycin resistant enterococci, ESBL: extended spectrum beta lactamase, MRSA: methicillin resistant Staphylococcus aureus, CDI: Clostridium difficile infection.

Table S2: Patient characteristics based on ceftazidime-avibactam therapy vs. standard of care (SOC)

Variable	CZA<br>81 (15.4)	SOC<br>269 (51.3)	Total<br>524 (100)	p-value
I. Demographics				
**Gender:**				0.52
Male	63 (67)	224 (63.5)	287 (64.2)	
Female	31 (33)	129 (36.5)	160 (35.8)	
Age	72 ± 13	58 ± 14	62 ± 14	0.06
**Ward:**				0.105
• Medical ward	23 (24.5)	125 (35.4)	148 (33.1)	
• Surgical ward	36 (38.3)	126 (35.7)	162 (36.2)	
• Intensive care unit	35 (37.2)	102 (28.9)	137 (30.6)	
ICU vs. other wards	36 (38.3)	102 (28.9)	138 (30.9)	0.079
Length of stay	44 ± 22	115 ± 89	79 ± 55	**0.005**
II. Clinical course				
**Comorbidities:**				
• Congestive heart failure	25 (26.9)	73 (20.7)	98 (22)	
• Chronic lung disease	26 (28)	68 (19.3)	94 (21.1)	0.2
• Liver disease	11 (25.6)	32 (9.1)	43 (9.6)	0.442
• Chronic Kidney Disease	27 (28.7)	83 (23.5)	110 (24.6)	0.297
• Metastatic malignancy	4 (4.3)	21 (5.9)	25 (5.6)	0.623
• Hematologic malignancy	6 (6.4)	38 (10.8)	44 (9.8)	0.205
• Diabetes mellitus	18 (22.8)	74 (21.1)	92 (21.4)	0.739
• Neutropenia	1 (1.3)	16 (4.6)	17 (4)	0.333
• Solid Organ Transplant	22 (23.2)	36 (3.5)	58 (12.9)	**<0.001**
• HIV	0	2 (0.6)	2 (0.6)	> 0.99
Surgery during hospitalization				0.390
• Surgery not abdominal	19 (25)	115 (32.8)	134 (31.4)	
• Abdominal surgery	20 (26.3)	77 (21.9)	97 (22.7)	
Charlson score	4.7 ± 2	4.5 ± 2	3.6 ± 2	0.884
**Invasive Procedures:**				

• CVC	7 (7.4)	53 (15)	60 (13.4)	**0.056**
• Mechanical Ventilation	50 (53.2)	178 (50.4)	228 (51)	0.633
• Urinary catheter	67 (72)	278 (78.8)	345 (77.4)	0.169
• ECMO	8 (8.6)	26 (7.4)	34 (7.6)	0.689
• Hemodialysis	18 (19.1)	20 (5.7)	38 (8.5)	**< 0.001**
• CVVH	14 (15.1)	63 (17.8)	77 (17.3)	0.526
**Possible source:**				0.798
• Respiratory	17 (21.8)	74 (21.1)	91 (21.2)	
• CVC	22 (28.2)	78 (22.8)	100 (23.3)	
• Urinary catheter	8 (10.3)	29 (8.3)	37 (8.6)	
• Intraabdominal	7 (9)	41 (11.7)	48 (31)	
• Multiple sites	5 (6.4)	26 (7.4)	31 (7.2)	
• Unidentified	19 (24.4)	103 (9.3)	122 (28.4)	
Source control	26 (32.9)	105 (29.9)	131 (30.5)	0.785
III. Microbiological characteristics:				
Time to KPC BSI	24 ± 16	27 ± 20	28 ± 30	0.068
KPC-Kp colonization	65 (82.3)	159 (45.3)	224 (52.1)	**<0.001**
**KPC in samples:**				
• Respiratory	37 (39.4)	118 (33.4)	155 (34.7)	0.283
• Urinary	24 (25.3)	91 (25.8)	115 (25.7)	0.919
• Drainage	8 (8.5)	51 (14.4)	59 (13.2)	0.131
**MDR Coloniztion:**	12 (12.8)	15 (4.2)	27 (6)	**0.002**
• A.baumannii	12 (12.8)	7 (2)	19 (4.3)	**<0.001**
• VRE	1 (1.1)	6 (1.7)	7 (1.6)	> 0.99
CDI pre	5 (5.3)	8 (2.3)	13 (2.9)	0.118
**Polymicrobial BSI:**	5 (5.3)	36 (10.2)	41 (9.2)	0.145
• P.aeruginosa	0	5 (1.4)	5 (1.4)	0.589
• VRE	1 (1.1)	6 (1.7)	7 (1.6)	> 0.99
• ESBL	2 (2.1)	9 (2.5)	11 (2.5)	> 0.99
• MRSA	0	4 (1.1)	4 (1.1)	0.302
• Candida	1 (1.1)	5 (1.4)	6 (1.3)	> 0.99
**Post BSI:**				

• Candedemia	3 (3.2)	4 (1.1)	7 (1.6)	0.158
**General combination**	1 ± 1	2 ± 1	2 ± 1	0.538
**Empiric therapy:**	52 (55.9)	250 (71)	302 (67.9)	**0.006**
• Empiric therapy appropriate	32 (56.1)	115 (45.5)	147 (47.4)	0.144
• Days Empiric therapy	7 ± 9	13 ± 10	11 ± 10	0.815
• Combination empiric	1 ± 1	2 ± 1	2 ± 1	0.696
**Targeted therapy:**	84 (92.3)	263 (74.5)	347 (78.2)	**<0.001**
• Days Targeted Therapy	8 ± 4	10 ± 7	15 ± 13	0.187
• Combination targeted	1 ± 1	2 ± 1	2 ± 1	0.439
On-time therapy	63 (70.8)	214 (61)	277 (63)	0.087
**7-day nephrotoxicity:**				0.155
• nephrotoxicity	15 (34.9)	59 (25.7)	74 (27.1)	
• Acute renal failure at baseline or death before 7 days	13 (30.2)	54 (23.5)	67 (24.5)	
Time to death	17 ± 10	25 ± 12	21 ± 26	0.449
In-hospital mortality	28 (29.5)	147 (41.6)	175 (39.1)	**0.031**
30-day mortality	26 (27.4)	127 (36)	153 (34.2)	0.116

ICU: intensive care unit, SOT: solid organ transplant, HIV: Human immunodeficiency virus, CVC: central venous catheter, ECMO: extracorporeal membrane oxygenation, CVVH: continuous veno-venous hemofiltration , KPC: Klebsiella pneumoniae carbapenemase, VRE: vancomycin resistant enterococci, ESBL: extended spectrum beta lactamase, MRSA: methicillin resistant Staphylococcus aureus, CDI: Clostridium difficile infection.
